# Infant Formula Supplemented With Milk Fat Globule Membrane, Long-Chain Polyunsaturated Fatty Acids, and Synbiotics Is Associated With Neurocognitive Function and Brain Structure of Healthy Children Aged 6 Years: The COGNIS Study

**DOI:** 10.3389/fnut.2022.820224

**Published:** 2022-03-09

**Authors:** Ana Nieto-Ruiz, José A. García-Santos, Juan Verdejo-Román, Estefanía Diéguez, Natalia Sepúlveda-Valbuena, Florian Herrmann, Tomás Cerdó, Roser De-Castellar, Jesús Jiménez, Mercedes G. Bermúdez, Miguel Pérez-García, M. Teresa Miranda, M. Carmen López-Sabater, Andrés Catena, Cristina Campoy

**Affiliations:** ^1^Department of Paediatrics, School of Medicine, University of Granada, Granada, Spain; ^2^Instituto de Investigación Biosanitaria (ibs.GRANADA), Health Sciences Technological Park, Granada, Spain; ^3^EURISTIKOS Excellence Centre for Paediatric Research, Biomedical Research Centre, University of Granada, Granada, Spain; ^4^Department of Personality, Assessment & Psychological Treatment, School of Psychology, University of Granada, Granada, Spain; ^5^Nutrition and Biochemistry Department, Faculty of Sciences, Pontificia Universidad Javeriana, Bogotá, Colombia; ^6^Carlos III Health Institute, Madrid, Spain; ^7^Laboratorios Ordesa, Sant Boi de Llobregat, Spain; ^8^Mind, Brain and Behavior Research Centre—CIMCYC, University of Granada, Granada, Spain; ^9^Department of Biostatistics, School of Medicine, University of Granada, Granada, Spain; ^10^Department of Nutrition, Food Sciences and Gastronomy, Faculty of Pharmacy and Food Sciences, University of Barcelona, Barcelona, Spain; ^11^Institut de Recerca en Nutrició i Seguretat Alimentària de la UB (INSA-UB), Barcelona, Spain; ^12^National Network of Research in Physiopathology of Obesity and Nutrition (CIBERobn), Institute of Health Carlos III (Barcelona's Node), Madrid, Spain; ^13^Department of Experimental Psychology, School of Psychology, University of Granada, Granada, Spain; ^14^National Network of Research in Epidemiology and Public Health (CIBERESP), Institute of Health Carlos III (Granada's Node), Madrid, Spain

**Keywords:** neuroimaging, early nutrition, infant formula, breastfeeding, MFGM, LC-PUFAs, synbiotics, cognition

## Abstract

**Background:**

Adequate nutrient intake during the first few months of life plays a critical role on brain structure and function development.

**Objectives:**

To analyze the long-term effects of an experimental infant formula (EF) on neurocognitive function and brain structure in healthy children aged 6 years compared to those fed with a standard infant formula or breastfed.

**Methods:**

The current study involved 108 healthy children aged 6 years and participating in the COGNIS Study. At 0–2 months, infants were randomized to receive up to 18 months of life a standard infant formula (SF) or EF enriched with milk fat globule membrane (MFGM), long-chain polyunsaturated fatty acids (LC-PUFAs) and synbiotics. Furthermore, a reference group of breastfed (BF) infants were also recruited. Children were assessed using neurocognitive tests and structural Magnetic Resonance Imaging (MRI) at 6 years old.

**Results:**

Experimental infant formula (EF) children showed greater volumes in the left orbital cortex, higher vocabulary scores and IQ, and better performance in an attention task than BF children. EF children also presented greater volumes in parietal regions than SF kids. Additionally, greater cortical thickness in the insular, parietal, and temporal areas were found in children from the EF group than those fed with SF or BF groups. Further correlation analyses suggest that higher volumes and cortical thickness of different parietal and frontal regions are associated with better cognitive development in terms of language (verbal comprehension) and executive function (working memory). Finally, arachidonic acid (ARA), adrenic acid (AdA), docosahexaenoic acid (DHA) levels in cheek cell glycerophospholipids, ARA/DHA ratio, and protein, fatty acid, and mineral intake during the first 18 months of life seem to be associated with changes in the brain structures at 6 years old.

**Conclusions:**

Supplemented infant formula with MFGM components, LC-PUFAs, and synbiotics seems to be associated to long-term effects on neurocognitive development and brain structure in children at 6 years old.

**Clinical Trial Registration:**

https://www.clinicaltrials.gov/, identifier: NCT02094547.

## Introduction

Proteins, long–chain polyunsaturated fatty acids (LC-PUFAs), iron, zinc, iodine, and B vitamins play a critical role on brain function and structure development (1–3). Nevertheless, given that the infant brain evolves from early childhood to adolescence, early-life nutritional deficiencies may have negative long-lasting or permanent effects on later cognitive function (4), including impairment on visuo-perceptual functions (5), working memory (6, 7), language (8), and executive functions (9).

It is well-established that breastfeeding is related to optimal brain maturation (10, 11) and neurodevelopment (12) in life. When breastfeeding is not possible, infant formulas are available to satisfy infant's nutritional and energy requirements. However, their functional and nutritional properties vary considerably from breast milk, and new bioactive compounds are being added to narrow the functional and nutritional gap with breast milk (13). In this regard, supplementation of infant formula with LC-PUFAs, mainly with docosahexaenoic and arachidonic acids (DHA and ARA, respectively), seems to be related with short and long-term beneficial effects on cognitive and visual development (14), learning, vocabulary, intelligence (15), processing information (16), and attention and inhibition systems (17). Other studies, however, have shown no long-term effects of LC-PUFA-enriched infant formula on neurodevelopmental and psychomotor outcomes (18, 19). On the other hand, infant formula supplementation with milk fat globule membrane (MFGM) has also gained interest due to its content in bioactive components and complex polar lipids that might have a potential role on infant neurodevelopment (20–22). Furthermore, it is important to notice that synbiotics (pre-plus probiotics) are also being added to infant formulas to ensure a healthy establishment of gut microbiota and communication along the microbiota-gut-brain axis, thus modulating neurodevelopment, brain function, and behavior throughout the life span (23, 24). However, the combined long-term effects of all these bioactive components are not well-established, and further studies are still needed to clarify the role of early nutrition based on bioactive compound-enriched infant formula on brain structure and cognitive function later in life.

Having in mind these considerations, the aim of this study was to analyze the long-term effects of an infant formula supplemented with MFGM, LC-PUFAs, and synbiotics on neurocognitive function and brain structure in healthy children aged 6 years compared to those who received a standard infant formula or breastfeeding.

## Materials and Methods

### Ethics, Informed Consent, and Permissions

The COGNIS study was conducted according to the updated principles of the Declaration of Helsinki II (25, 26), and was approved by the Research Bioethical Committee from the University of Granada (Spain), the Bioethical Committees for Clinical Research from San Cecilio University Clinical Hospital, and University Mother-Infant Hospital of Granada (Spain). Prior to involving each child in the study, families were informed about study procedures and a signed written informed consent was obtained from each parent, legal guardian, or caregiver.

### Study Design and Subjects

The current analysis included 108 children aged 6 years and participating in the COGNIS Project, a prospective, randomized, and double-blind study consisting in a nutritional intervention based on an infant formula supplemented with bioactive nutrients (registered at https://clinicaltrials.gov/ct2/show/NCT02094547?term=NCT02094547&draw=2&rank=1, Identifier: NCT02094547). Detailed information on this project, including study design, subject recruitment, and population characteristics, have been previously described (27, 28). Originally, a total of 220 full-term healthy Spanish infants were included in the study. One hundred seventy infants, aged between 0 and 2 months old, were randomized (ratio 1:1) to receive, during their first 18 months of life, either a standard infant formula (SF: *n* = 85) or an experimental infant formula (EF: *n* = 85) enriched with MFGM components [10% of total protein content (wt:wt)], synbiotics [Fructooligosaccharides (FOS): Inulin proportion 1:1; *Bifidobacterium longum* subsp *infantis* CECT7210 (*Bifidobacterium infantis* IM1) and *Lactobacillus rhamnosus* LCS-74], LC-PUFAs (including DHA and ARA), gangliosides, nucleotides, and sialic acid. A full description of the nutritional composition of both infant formulas has been previously reported (28). Infants received initiation formula up to 6 months of age and the corresponding follow-on formula between 6 and 18 months of age. Additionally, 50 exclusively breastfed infants (BF) for at least 2 months were enrolled between 0 and 6 months old as a reference group.

**Figure 1:**
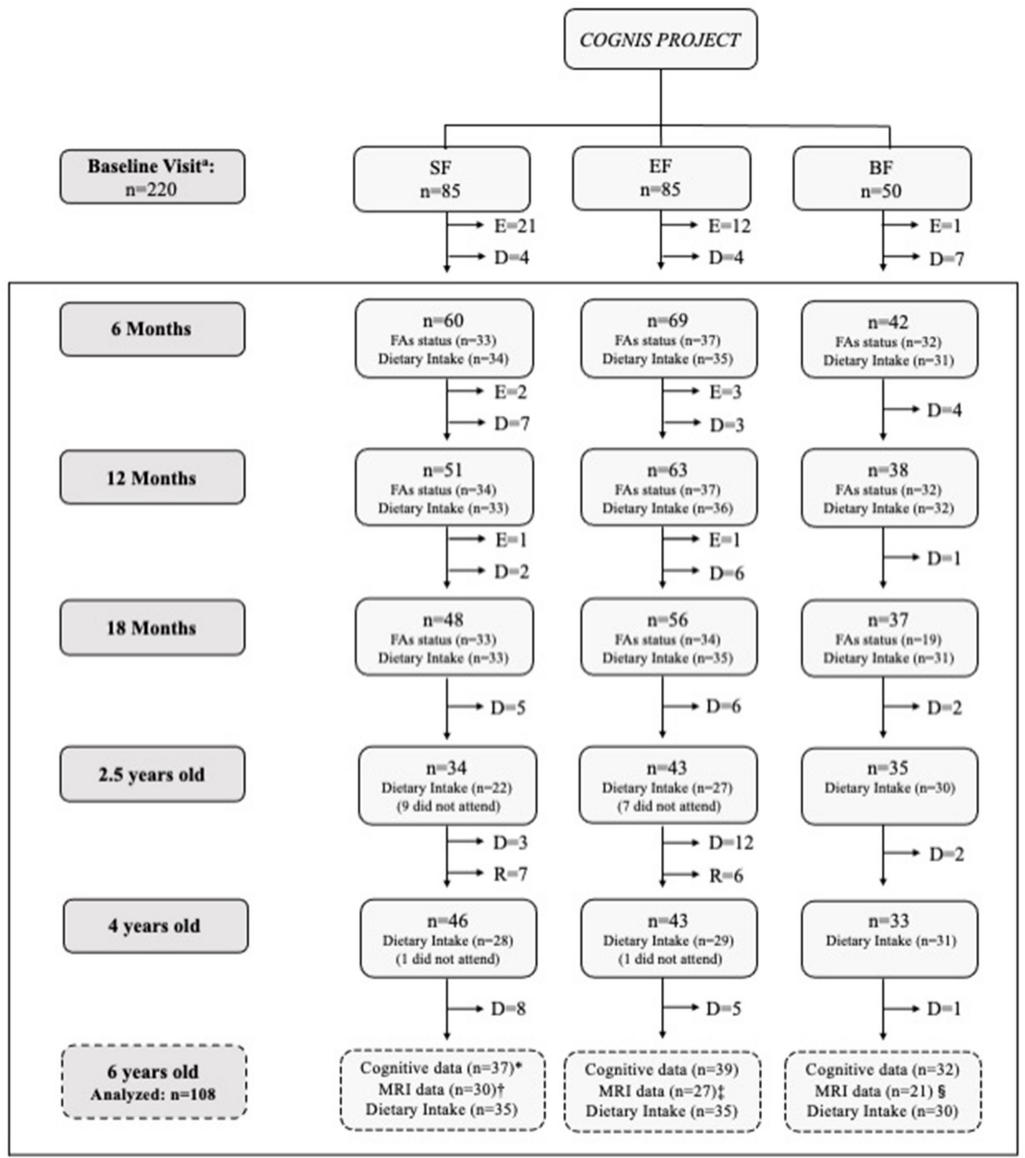
Participant flow-chart from baseline visit to 6 years old. BF, Breastfed infants; D, Drop-outs; E, Exclusions; EF, Experimental infant formula; MRI, Magnetic Resonance Imaging; *n*, Sample size; R, Recovered; SF, Standard infant formula. Fatty acid (FA) status was analyzed from available cheek cell samples of children who attended to 6 years follow-up visit. Incomplete dietary intake data were excluded from the current analysis. At 6 years old: *2 children did not attend to cognitive assessment. Some MRI data from participants (*n* = 30) were eliminated because the quality of the brain images was not adequate to be analyzed due to excessive movement of children inside the scanner: Unable to analyze the data from 7 children; ^‡^Unable to analyze the data from 14 children; ^§^Unable to analyze the data from 11 children. ^a^BF infants were randomized between 0–6 months of age.

A detailed participant's flowchart from the baseline visit to 6 years old is shown in Figure 1. Up to 18 months of life, a total of 40 infants had to be excluded in the formula groups as follows: 24 were excluded in the SF group (1 infant due to perinatal hypoxia, 1 infant had formula non-related growth deficiency, 15 infants did not take the infant formula, 2 had infantile colic, 3 were excluded due to lactose intolerance, 1 infant due to digestive surgical intervention, and 1 infant suffered hydrocephalus); 16 infants were excluded in the EF group (2 infants presented formula non-related growth deficiency, 2 infants lactose intolerance, 11 infants did not take the infant formula, and 1 was excluded due to epileptic seizure). Furthermore, one infant from the BF group was excluded because he was not exclusively breastfed for at least 2 months. During the follow-up visits, some participants decided to drop out of the study. Afterwards, 108 children attended the follow up call at 6 years old, and their neurocognitive performance was evaluated using the Kaufman Brief Intelligence Test (K-BIT) (29), the Oral Language Test of Navarra Revised (PLON-R) (30), and the Computerized Battery for Neuropsychological Evaluation of Children (BENCI) (31) (SF: *n* = 37; EF: *n* = 39; BF: *n* = 32). All participants were also invited to participate in a Magnetic Resonance Imaging (MRI) session. However, some data from participants (*n* = 30) were taken out of current analysis due to poor image quality caused by the excessive motion of children during MRI acquisition. Therefore, neuroimage data were finally obtained from 78 children aged 6 years (SF: *n* = 30; EF: *n* = 27; BF: *n* = 21). Furthermore, nutritional information from participants in the COGNIS study was also collected, including fatty acid (FA) status up to 18 months of age (SF: 33; EF: 34; BF: 31) and complete dietary intake up to 6 years old (SF: 35; EF: 35; BF: 30).

### Data Collection and Assessment

Baseline information regarding parents was obtained upon study entry, including age, pre-conceptional maternal body mass index (BMI), gestational weight gain (GWG), smoking during pregnancy, educational level, place of residence, employment, and socioeconomic status. Postpartum depression was evaluated with the Spanish version of the Edinburgh Postnatal Depression Scale (32). Parents' intelligence quotient (IQ) was assessed using the G factor of the Cattel Intelligence test (33, 34).

Baseline characteristics of infants (gestational age, type of delivery, sex, siblings, and timing of breastfeeding) were collected using questionnaires and medical records. In addition, child anthropometric parameters such as BMI, head circumference, and waist circumference were also registered at 6 years of age.

#### Fatty Acid Status

Analysis of FA status was performed from cheek cell samples collected at 3, 6, 12, and 18 months of life, as previously reported (35). This method is a valid index of essential fatty acid status as it can be monitored frequently and is reported to be associated with functional parameters in infants (36). In addition to the easy and non-invasive nature of this technique, cheek cell fatty acids may serve as a marker of the essential fatty acid content, especially of DHA and ARA, in plasma, tissue concentrations (red blood cells), and the diet (37). Briefly, samples from the inside of the infants' cheeks were collected 1 h after feeding using a Rovers^®^ EndoCervex-Brush^®^ supplied by Deltalab (Barcelona, Spain), and cell pellets were obtained by centrifugation and stored at −80°C until further analysis. Cheek cell glycerophospholipid fraction was isolated using methanol with butylated hydroxytoluene (BHT), while FA methyl esters were obtained using sodium methylate in methanol (25 wt% in methanol) and boron trifluoride methanol solution (14% v/v). Rapid gas chromatography was used to separate FAs in cheek cell samples (35, 38), and quantification was done by normalization. Lastly, the results were expressed in relative amounts (percentage).

#### Dietary Intake

Participants' dietary intake was evaluated at 6, 12, and 18 months and 2.5, 4, and 6 years old by a quantitative 3-day dietary record based on the methods for food monitoring and nutrient intake indicated by Food and Agriculture Organization of the United Nations (FAO) (39). DIAL software (Alce Ingeniería, Madrid, Spain) (40) was used to analyze dietary records, which converts food consumption data into nutrient intake (macro- and micronutrients, including FAs profile) in accordance to a previously described methodology (41).

#### Neurocognitive Evaluation

##### Kaufman Brief Intelligence Test

The Spanish version of K-BIT (29) was used to evaluate verbal and non-verbal intelligence through two subtests: *vocabulary and matrices*. In the *vocabulary* subtest, children observed a series of pictures and named the object presented on them. *Matrices* subtest is a measure of abstract reasoning. In this case, the child selected a picture or abstract design that best completes a visual pattern following a visual analogy. In both subtests, the dependent variable was the number of correct responses. Furthermore, the K-BIT test also provides a general IQ based on the sum of scores obtained in the *vocabulary* and *matrices* subtests. According to K-BIT test standards, the normal range of typical scores is between 85 and 115 points (29, 42).

##### Oral Language Test of Navarra Revised (PLON-R): Language Assessment

Oral Language Test of Navarra Revised (PLON-R) is a standardized test that allows an early detection or screening of the oral language development in children aged between 3 and 6 years old. This test is not only focused on the language dimensions (form, content, and use) with specific activities for each dimension, but also provides a total punctuation on language development. The scores of each one of the dimensions are transformed into typical scores. The normal score, according to PLON-R test standardized by age, is as follows: form = 65; content ≥ 62; use ≥ 52; total ≥ 51 (30).

##### Computerized Battery for Neuropsychological Evaluation of Children

Computerized Battery for Neuropsychological Evaluation of Children (BENCI) is a computerized tool that evaluates neuropsychological functions and neurodevelopmental domains in children, such as *immediate and delayed memory, attention, visual motor coordination, verbal fluency and comprehension, processing speed, and executive functions* (31, 43). All these tests were developed using valid neuropsychological procedures assumed from the literature of neuropsychological assessment, and were principally based on the NUTRIMENTHE Neuropsychological Battery (43, 44). In the current study, neurocognitive function was assessed using the following tasks (45):

-*Verbal Comprehension (images) (language)*. A combination of images of a given category (animals) was shown, and the children received instructions (auditory) through which they should select a given image that fulfills the indicated conditions (type of animal, position, type of activity that one can carry out, and/or color: e.g., “*Touch the frog that is next to the dog”*).
-*Continuous performance (sustained attention)*. Various blocks in a series of letters (100 essays) appeared on the screen and the child should touch the tablet screen each time the correct stimulus appears (an A after an X). The rest of the letters were used as distractor elements.
-*Semantic Fluency (executive function)*. This test indicates a category (for example, animals) and the child should respond, as fast as he/she can, with all the words he/she knows within the same category in 60 s.
-*Working Memory (executive function)*. The children listened to 8 series of number and color sequences. After each sequence, the child should separately repeat the numbers and the colors that he/she had heard in the same order.
-*Verbal Memory (short and long-term memory)*. At the beginning of the task, the child listened to 6 series of words, and should memorized as many as possible. After each sequence, the child should repeat all the words that he/she could remember. After 20 min, in the delayed recall essay, the child should repeat out loud all those words he/she could remember from the previous list. Finally, in the recognition essay, the child listened to a series of words, half of which were in the list above. The child answered “*yes”* or “*no”* to whether each word was in the list.
-*Simple Reaction Time Test (processing speed)*. This test required that the child to press any key as fast as possible every time a cross (+) appeared on the screen (50 essays).
-*Go/no-Go task (executive function: inhibition)*. During this test, two alternating elements (bear and dolphin) kept appearing on the screen. In the first phase of the test, the child should state the distinctive element of the two (bear) and touch the tablet screen when it appeared. After listening to a sound that represents the phase change, the distinctive element appeared to be the other (dolphin), to which the child should press the tablet screen when it appeared. The dependent measure was the total number of correct answers.

#### Magnetic Resonance Imaging Procedure

##### Imaging Data Acquisition

Prior to the neuroimaging session, children were familiarized with scanner's sounds and MRI environment using a mock MRI scanner. Brain data were acquired using a 3T MRI scanner equipped with a 32-channel phased-array head coil for reception (Magnetom Trio Siemens Medical System, ERLANGEN, Germany) located at Mind, Brain, and Behavior Research Center (CIMCYC, University of Granada, Spain). A high resolution T1-weighted 3D magnetization-prepared rapid gradient-echo (MPRAGE) sequence was acquired for each participant with the following parameters: Repetition Time (TR) = 2.3 ms, Echo Time (TE) = 3.1 ms, flip angle = 9°, Field of View (FOV) = 256 × 256 mm, matrix size=320 × 320, and number of slices=208, resulting in an isotropic resolution of 0.8 × 0.8 × 0.8 mm. Total acquisition time for the T1 sequence was 6 min and 35 s. Head movements were minimized using a foam system around the participant's head. Furthermore, a cartoon film was projected to reassure the child during the MRI scanning.

##### Neuroimage Processing

All images were visually inspected for major artifacts and realigned to the anterior commissure-posterior commissure (AC-PC) line. Image processing was performed using the automated processing “*recon-all”* pipeline in FreeSurfer software (version 6.0, http://surfer.nmr.mgh.harvard.edu/) on the Alhambra Cluster of the University of Granada (Spain). Preprocessing steps involved intensity normalization, registration to Talairach space, skull stripping, segmentation of white matter (WM), tessellation of the WM boundary, and automatic correction of topological defects. After that, cerebral cortex was parceled into regions of interest (ROIs) based on gyral and sulcal structures from the Destrieux atlas (46–49). FreeSurfer outputs were also visually inspected to check for correct segmentation and parcellation. Volumes and cortical thicknesses of brain parcellation based on the Destrieux atlas were extracted along with brain subcortical volumes.

### Statistical Analysis

All statistical analyses were performed using IBM^®^ SPSS Statistics^®^ program, version 22.0 (SPSS Inc. Chicago, IL, USA). Normally distributed variables were presented as mean and standard deviation (SD), and non-normal variables as median and interquartile range (IQR). Categorical variables were shown as frequencies and percentages. ANOVA or Welch test were performed for normally distributed variables, Kruskal Wallis test was performed for non-normal continuous variables, and Chi-square or Fisher test was performed for categorical variables. Normal distribution was tested using Kolmogorov-Smirnov and/or Shapiro Wilk test. We also used one-way ANOVAs to examine differences between SF, EF, and BF groups in neurocognitive tests scores, brain volumes, and cortical thicknesses. Total intracranial volume was included as covariate in all brain volume analyses. Moreover, to discard the differences that could be driven by confounders *(maternal age, familiar socioeconomic status, smoking during pregnancy, age, and sex*) (50–52), we performed additional analyses of group comparisons using a one-way analysis of covariance (ANCOVA) that included these confounders. In the event of significant group differences, Bonferroni-corrected *post-hoc* comparisons were used to identify significant pair-wise group differences. To explore whether the brain differences could be driven by the FA concentrations and dietary intake, we performed stepwise linear regression analyses, using one brain region as dependent variable and FA concentration, dietary intake, and the aforementioned confounders as independent variables. Finally, Pearson correlation analyses were performed to estimate the relationship between child neurocognitive performance and brain volumes and thickness of those regions that were statistically different between study groups. *p* < 0.05 were considered statistically significant.

## Results

### Parental and Child Characteristics of the Study Participants at 6 Years Old

A comparative analysis between study groups of parents' and children's baseline characteristics participating at 6 years old in the COGNIS follow-up study is shown in Table 1. Significant differences between study groups were found in parents' age, educational level, and in their socioeconomic status. In fact, BF mothers were older and showed higher educational level than mothers of SF and EF infants (*p* = 0.013 and *p* = 0.001, respectively). No significant differences were found between study groups in other maternal baseline characteristics related to child neurocognitive function and brain structure, including pre-conceptional BMI (pBMI), GWG, and smoking during pregnancy. Table 1 also shows that fathers from the BF group were older compared to fathers of SF infants (*p* = 0.033) and presented higher educational level than EF-fed infants' fathers (*p* = 0.005). Concerning socioeconomic status, those parents whose children were breastfed had higher status compared to both SF- and EF-fed infants' parents (*p* = 0.002).

**Table 1:** Baseline characteristics of parents and children participating in the COGNIS study depending on their type of feeding during infancy^1^.

		**SF (*n* = 37)**	**EF (*n* = 39)**	**BF (*n* = 32)**	***p*** **^2^**
**Mother**	
Age (years)		31.5 (10.8)^a^	30.0 (5.0)^a^	34.0 (8.5)^b^	**0.013**
pBMI (kg/m^2^)		24.5 ± 4.2	25.6 ± 4.3	24.6 ± 2.8	0.468
GWG (kg)		5.8 ± 5.2	6.7 ± 4.8	6.4 ± 3.4	0.723
Type of delivery	Vaginal	28 (75.7)	28 (71.8)	24 (75.0)	0.919
	Cesarean section	9 (24.3)	11 (28.2)	8 (25.0)	
IQ (points)		104.0 (16.5)	100.0 (19.0)	111.0 (21.0)	0.105
Educational level	NS/Primary	4 (10.8)^a,b^	12 (30.9)^a^	2 (6.3)^b^	**0.001**
	Secondary	10 (27.0)	10 (25.6)	2 (6.3)	
	VT	14 (37.8)	10 (25.6)	9 (28.1)	
	University/Ph.D	9 (24.4)^a^	7 (17.9)^a^	19 (59.4)^b^	
Postpartum depression	No	29 (78.4)	32 (84.2)	27 (84.4)	0.749
Smoking during pregnancy	No	30 (81.1)	33 (84.6)	30 (93.8)	0.299
Employment status	Unemployed	9 (24.3)	5 (12.8)	5 (15.6)	0.525
	Domestic work	1 (2.7)	3 (7.7)	1 (3.1)	
	Temporary contract	2 (5.4)	7 (17.9)	4 (12.5)	
	Stable employment	25 (67.6)	24 (61.5)	22 (68.8)	
**Familiar**	
Socioeconomic status	Low	7 (18.9)	8 (20.5)	1 (3.1)	**0.002**
	Middle-Low	18 (48.6)	19 (48.7)	8 (25.0)	
	Middle-High	10 (27.0)	10 (25.6)	13 (40.6)	
	High	2 (5.4)^a^	2 (5.1)^a^	10 (31.3)^b^	
Place of residence	Urban	14 (37.8)	11 (28.2)	6 (18.8)	0.216
	Rural	23 (62.2)	28 (71.8)	26 (81.3)	
Siblings	0	8 (21.6)	11 (28.2)	5 (15.6)	0.445
	≥1	29 (78.4)	28 (71.8)	27 (84.4)	
**Father**	
Age (years)		32.3 ± 7.0^a^	33.2 ± 6.1^a,b^	36.2 ± 4.5^b^	**0.033**
Educational level	NS/Primary	8 (21.6)^a,b^	17 (43.6)^a^	5 (15.6)^b^	**0.005**
	Secondary	18 (48.6)	10 (25.6)	7 (21.9)	
	VT	5 (13.5)	8 (20.5)	8 (25.0)	
	University/Ph.D	6 (16.3)^a,b^	4 (10.3)^a^	12 (37.5)^b^	
IQ (points)		106.8 ± 13.9	104.7 ± 16.2	106.5 ± 13.0	0.822
Employment status	Unemployed	5 (13.5)	4 (11.1)	1 (3.1)	0.302
	Domestic work	0 (0.0)	0 (0.0)	0 (0.0)	
	Temporary contract	6 (16.2)	2 (5.6)	3 (9.4)	
	Stable employment	26 (70.3)	30 (83.3)	28 (87.5)	
**Neonate**	
Gestational age (weeks)		40.0 (2.0)	40.0 (2.0)	40.0 (2.8)	0.826
Breastfeeding lactation (days)		13 (21.0)^a^	12 (31.0)^a^	450 (240.0)^b^	**<0.001**
Sex	Boy	24 (64.9)	25 (64.1)	13 (40.6)	0.073
	Girl	13 (35.1)	14 (35.9)	19 (59.4)	
**Children**	
Age (days)		2225 (38)	2222 (42)	2221.5 (26)	0.565
BMI (kg/m^2^)		16.1 (2.4)	16.6 (2.6)	15.8 (2.1)	0.308
Head circumference (cm)		51.5 ± 1.8	51.8 ± 1.6	51.6 ± 1.1	0.797
Waist circumference (cm)		53.9 (4.7)	55.2 (5.9)	53.9 (6.5)	0.356

1 *Data are presented as means ± SD for parametrically distributed data, n (%) for categorical data, and medians (IQRs) for non- parametrically distributed data. ^2^P-values for overall differences between COGNIS-groups. ANOVA test for normally distributed variables, Kruskal–Wallis test for non-normal continuous variables, and Chi-square or Fisher test for categorical variables. Values not sharing the same suffix (a,b) were significantly different in the Bonferroni post-hoc test. P < 0.05 are highlighted in bold. SF, Standard infant formula; EF, Experimental infant formula; BF, Breastfed infants; pBMI, pre-conceptional body mass index; GWG, Gestational Weight Gain; NS, No schooling; IQ, Intelligence quotient; VT, Vocational training; BMI, Body mass index*.

All infants participating in the COGNIS study were more frequently born by vaginal delivery, and no significant differences were found for characteristics at birth among study groups. All children were born at term and with birth weight adequate for gestational age. However, as expected, due to the COGNIS study design, days of breastfeeding significantly differed between formula (SF and EF) and BF groups (*p* < 0.001). Finally, at 6 years old, children from the three study groups did not differ in their anthropometric characteristics, including BMI and head and waist circumferences.

### Type of Early Feeding and Neurocognitive Function in Children Aged 6 Years

First, we analyze the potential long-term effects of the type of early feeding received during the first 18 months of life (EF vs. SF vs. BF) on later neurocognitive function at 6 years, comparing the K-BIT, PLON-R, and BENCI scores between the three COGNIS groups (Table 2). In the adjusted model, controlling for *maternal age, smoking during pregnancy, familiar socioeconomic status, and sex*, EF children showed higher IQ and vocabulary standard score in the K-BIT test than BF children (*p*_*adj*_ = 0.031, and *p*_*adj*_ = 0.022, respectively). Moreover, both SF and EF children presented less errors of commission in continuous performance task from BENCI battery (*p*_*adj*_ = 0.001) in comparison to BF children (Table 2). No significant differences in child language dimensions (PLON-R Test) at 6 years old were found between the three groups (Table 2).

**Table 2:** Children's neurocognitive function at 6 years old depending on the type of feeding during infancy^1^.

	**SF (*n* = 37)**	**EF (*n* = 39)**	**BF (*n* = 32)**	***p*** ^2^	***p*** **_adj_**
**Kaufman brief intelligence test (K-BIT)**					
Vocabulary (typical score)	107.91 ± 13.44^a,b^	113.16 ± 13.31^a^	103.73 ± 14.26^b^	0.177	**0.022**
Matrices (typical score)	111.51 ± 12.07	113.21 ± 12.00	111.78 ± 11.07	0.798	0.113
IQ (typical score)	109.64 ± 11.67^a,b^	113.46 ± 11.50^a^	105.54 ± 12.40^b^	0.302	**0.031**
**Oral language test of Navarra-Revised (PLON-R)**					
Form (typical score)	46.92 ± 17.30	47.46 ± 14.89	49.84 ± 12.61	0.702	0.991
Content (typical score)	54.95 ± 24.65	55.38 ± 15.23	60.34 ± 19.87	0.468	0.886
Use (typical score)	52.65 ± 23.01	53.95 ± 21.38	61.00 ± 16.38	0.207	0.896
Total score PLON-R (typical score)	55.78 ± 27.34	55.31 ± 20.33	63.34 ± 19.42	0.201	0.889
**Computerized battery for neuropsychological evaluation of children (BENCI)**					
Verbal comprehension (successes)	10.65 ± 2.06	11.16 ± 1.55	11.26 ± 1.86	0.327	0.237
Continuous performance (successes)	53.92 ± 4.72	51.17 ± 10.00	50.06 ± 8.51	0.055	0.115
Continuous performance (errors of commission)	8.14 ± 7.87^a^	9.44 ± 12.02^a^	18.35 ± 17.43^b^	**0.003**	**0.001**
Semantic fluency (successes)	7.65 ± 2.70	7.39 ± 2.11	8.06 ± 2.39	0.518	0.927
Working memory (successes)	4.00 ± 1.43	4.05 ± 1.39	4.00 ± 1.55	0.984	0.256
Verbal short-term memory (successes)	4.39 ± 1.10^a,b^	4.19 ± 0.95^a^	4.89 ± 0.73^b^	**0.010**	0.238
Verbal long-term memory (successes)	4.41 ± 1.76	3.95 ± 2.10	4.68 ± 1.58	0.251	0.263
Verbal long-term memory (recognition successes)	10.57 ± 1.88	10.97 ± 2.02	11.13 ± 1.12	0.387	0.525
Reaction time (ms)	542.92 ± 54.25	557.55 ± 64.87	553.42 ± 151.25	0.569	0.828
Go/no-Go task (successes)	46.57 ± 4.50	48.28 ± 6.80	48.13 ± 5.73	0.308	0.335
Go/no-Go task (errors of commission)	3.24 ± 2.50	3.03 ± 2.45	3.94 ± 2.32	0.283	0.071

1 *Data are presented as means ± SD*.

2 *P-values for differences between COGNIS-groups. ANOVA test for normally distributed variables*.

*P_adj_ are univariate analysis of covariance (ANCOVA) adjusted by maternal age, smoking during pregnancy, familiar socioeconomic status and sex of the children. Values not sharing the same suffix (a,b) were significantly different in the Bonferroni post-hoc test. P < 0.05 are highlighted in bold*.

*SF, Standard infant formula; EF, Experimental infant formula; BF, Breastfed infants; ms, milliseconds*.

### Analysis of Brain Volume and Cortical Thickness in Children Aged 6 Years and Effects of Early Nutrition

**Figure 2:**
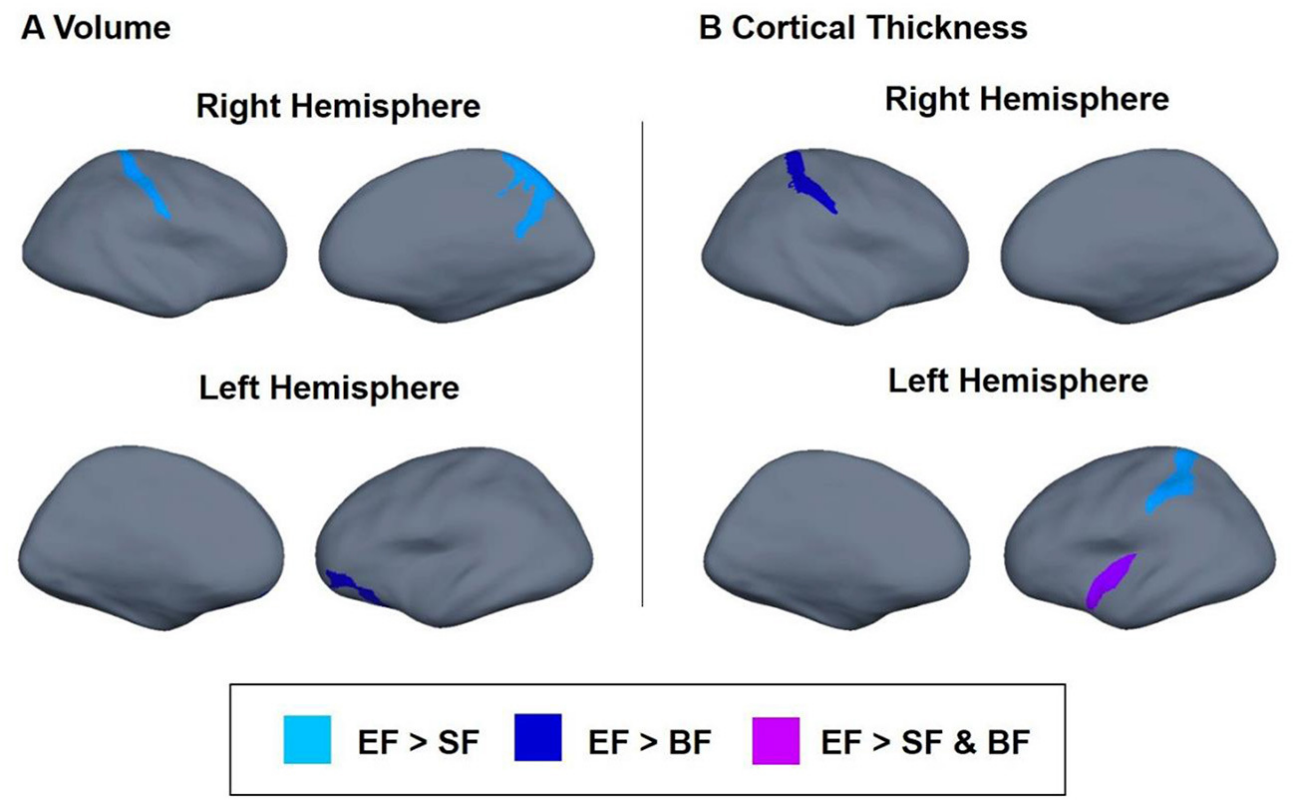
Differences in brain volume **(A)** and cortical thickness **(B)** between children participating in the COGNIS study at 6 years of life. Analysis adjusted by smoking during pregnancy, maternal age, familiar socioeconomic status, age, and sex of the children. Brain volume analysis was also corrected for total brain volume. Experimental infant formula (EF) > Standard infant formula (SF) (light blue); EF > BF (dark blue); and EF > SF and EF > BF (purple). SF, Standard infant formula; EF, Experimental infant formula; BF, breastfed infants.

Next, we tested whether the type of early nutrition during the first 18 months of life *(SF vs. EF vs. breastfeeding)* had any effects on later brain structure, brain volume, and cortical thickness. Significant results, adjusted by *smoking during pregnancy, maternal age, familiar socioeconomic status, age, and sex (plus total brain volume for volume brain analysis)*, are presented in Table 3. Children fed with EF presented higher volumes in parietal regions than the SF children (*p*_*adj*_ = 0.002), particularly, in the right postcentral gyrus (*p*_*adj*_ = 0.015) and in the right precuneus (*p*_*adj*_ = 0.009). EF children also showed higher left orbital volume than BF children (*p*_*adj*_ = 0.012). Regarding cortical thickness, adjusted analyses showed that EF children presented higher thicknesses in the left inferior circular insular sulcus compared to SF and BF children (*p*_*adj*_ = 0.012). Moreover, children fed with EF presented increased cortical thickness in the left occipito-temporal sulcus compared to the SF group (*p*_*adj*_ = 0.027), and in the right postcentral sulcus compared to the BF group (*p*_*adj*_ =0.017). Taken together, these results suggest a greater volume of parietal regions and higher cortical thickness in EF children aged 6 years than SF children (Figure 2, light blue), BF children (Figure 2, dark blue), or both (Figure 2, purple).

**Table 3:** Differences in brain volume and thickness between 6 years old children fed with standard infant formula (SF), experimental infant formula (EF), or breastfed (BF) during their first 18 months of life^1^.

**Brain region**	**SF (*n* = 30)**	**EF (*n* = 27)**	**BF (*n* = 21)**	***P*** ^2^	***p*** **_adj_**
**Volume (mm** ^**3**^ **)**
Right parietal	71,562.67 ± 7,999.80^a^	77,167.15 ± 8,496.22^b^	73,602.10 ± 7,684.33^b^	**<0.001**	**0.002**
Right postcentral gyrus	4,258.53 ± 633.89^a^	4,885.78 ± 911.37^b^	4,429.24 ± 797.78^a,b^	**0.008**	**0.015**
Right precuneus	7,144.43 ± 1,201.38^a^	7,856.89 ± 1,164.59^b^	7,283.62 ± 974.12^a,b^	**0.029**	**0.009**
Left orbital	3,071.83 ± 503.06^a,b^	3,098.48 ± 535.85^a^	2,628.38 ± 554.63^b^	**0.006**	**0.012**
**Cortical thickness (mm)**
Left inferior circular insular sulcus	3.08 ± 0.17^b^	3.21 ± 0.20^a^	3.069 ± 0.17^b^	**0.010**	**0.012**
Left occipito-temporal sulcus	2.69 ± 0.15^a^	2.78 ± 0.12^b^	2.734 ± 0.15^a,b^	**0.047**	**0.027**
Right postcentral sulcus	2.46 ± 0.13^a^	2.53 ± 0.13^b^	2.423 ± 0.12^a,b^	**0.013**	**0.017**

1 *Data are means ± SD*.

2 *P-values for differences between the COGNIS-groups. ANOVA test for normally distributed variables. Volume analysis is corrected by total brain volume*.

*P_adj_ are univariate analysis of covariance (ANCOVA) adjusted by smoking during pregnancy, maternal age, familiar socioeconomic status, age and sex of the children. Values not sharing the same suffix (a,b) were significantly different in the Bonferroni post-hoc test. P < 0.05 are highlighted in bold*.

*SF, Standard infant formula; EF, Experimental infant formula; BF, Breastfed infants*.

### Associations Between Brain Structure and Neurocognitive Function in Children Aged 6 Years

Afterwards, we inquired whether there might be potential associations between child neurocognitive function at 6 years and brain structure (volume brain and cortical thicknesses), particularly in those regions in which significant differences in previous adjusted model analyses were found. In this regard, Pearson correlation analyses revealed that greater volume in the right parietal and right precuneus was positively associated with better verbal comprehension (*r* = 0.267, *p* = 0.019 and *r* = 0.278, *p* = 0.014, respectively), while better working memory was only positively related to higher right parietal volume (*r* = 0.257, *p* = 0.024; Table 4). In this line, our previous analysis showed that children fed with EF or BF presented greater volume in these regions (Table 3) and more successes, but without statistically significant differences in verbal comprehension and working memory in the BENCI Neuropsychological battery (Table 2). Furthermore, cortical thickness in the left occipito-temporal sulcus was positively correlated to better working memory (*r* = 0.245, *p* = 0.032; Table 4). It is important to note that children who were fed with EF showed significantly greater cortical thickness in this region (Table 3) and better scores in the abovementioned neuropsychological test (Table 2), although no statistically significant differences were found.

**Table 4:** Relationship between brain structure and neurocognitive developmental scores in children aged 6 years (*n* = 78)^a^.

		**Neurocognitive tasks**	
**Brain regions**		**Verbal comprehension (successes)**	**Working memory (successes)**
**Volume (cm** ^**3**^ **)**		
Right parietal	*r*	0.267	0.257
	*P* ^1^	**0.019**	**0.024**
Right precuneus	*r*	0.278	0.125
	*P* ^1^	**0.014**	0.277
**Cortical thickness (mm)**		
*Left occipito-temporal sulcus*	*r*	0.048	0.245
	*P* ^1^	0.680	**0.032**

a *P-values for correlations between brain structure and neurocognitive development. P < 0.05 are highlighted in bold*.

*r, Pearson correlation coefficient*.

### Infant's LC-PUFAs Status During the First 18 Months

To gain additional insight into the effects of early nutrition on brain structure, we analyzed FA levels of buccal cheek cell phospholipids in the COGNIS children at 6, 12, and 18 months of life, depending on their type of early feeding (Table 5). At 6 months of life, BF infants presented higher concentrations of ARA (*p* < 0.001), adrenic acid (AdA) (*p* < 0.001), and DHA (*p* < 0.001) in comparison to formula-fed infants. Moreover, as expected, both ARA and DHA concentrations were also increased in EF infants compared to SF infants. Nevertheless, we found higher concentrations of eicosapentaenoic acid (EPA) in EF infants than in SF infants (*p* < 0.001). AdA concentrations were also higher in the BF group compared to the EF infants (*p* = *0.0*02) at 12 months of life, while both BF and EF infants showed higher DHA levels than SF infants (*p* < 0.001). At 18 months of life, formula-fed infants presented higher ARA and AdA levels than BF infants (*p* = 0.002 and *p* < 0.001, respectively), and DHA concentrations were higher in the EF group compared to the SF group (*p* = 0.001). Finally, ARA/DHA index, which reflects both endogenous synthesis and exogenous supply, were lower in BF and EF infants compared to SF infants at 6, 12, and 18 months of life (*p* < 0.001; Table 5).

**Table 5:** Influence of type of early feeding on long-chain polyunsaturated fatty acids (LC-PUFAs) concentration in infants at 6, 12, and 18 months of life^1^.

**FAs concentrations (%)**	**SF**	**EF**	**BF**	***P*** ^2^
**6 months**	**(*****n*** **=** **33)**	**(*****n*** **=** **37)**	**(*****n*** **=** **32)**	
ARA	1.75 ± 0.56^a^	2.16 ± 0.63^b^	2.80 ± 0.59^c^	**<0.001**
AdA	0.22 ± 0.08^a^	0.21 ± 0.08^a^	0.29 ± 0.08^b^	**<0.001**
EPA	0.10 ± 0.05^a^	0.13 ± 0.06^b^	0.12 ± 0.05^a,b^	**0.027**
DHA	0.35 ± 0.19^a^	0.83 ± 0.31^b^	1.10 ± 0.33^c^	**<0.001**
ARA/DHA	5.52 ± 1.52^a^	2.79 ± 0.73^b^	2.72 ± 0.78^b^	**<0.001**
**12 months**	**(*****n*** **=** **34)**	**(*****n*** **=** **37)**	**(*****n*** **=** **32)**	
ARA	2.08 ± 0.56	2.13 ± 0.56	2.36 ± 0.59	0.101
AdA	0.24 ± 0.05^a,b^	0.21 ± 0.05^a^	0.27 ± 0.07^b^	**0.002**
EPA	0.12 ± 0.06	0.14 ± 0.06	0.12 ± 0.05	0.144
DHA	0.48 ± 0.18^a^	0.77 ± 0.25^b^	0.87 ± 0.30^b^	**<0.001**
ARA/DHA	4.59 ± 1.29^a^	2.90 ± 0.60^b^	2.86 ± 0.72^b^	**<0.001**
**18 months**	**(*****n*** **=** **33)**	**(*****n*** **=** **34)**	**(*****n*** **=** **19)**	
ARA	2.21 ± 0.55^a^	2.33 ± 0.56^a^	1.79 ± 0.46^b^	**0.002**
AdA	0.28 ± 0.07^a^	0.27 ± 0.06^a^	0.21 ± 0.05^b^	**<0.001**
EPA	0.13 ± 0.06	0.16 ± 0.07	0.15 ± 0.10	0.172
DHA	0.59 ± 0.20^a^	0.84 ± 0.29^b^	0.75 ± 0.33^a,b^	**0.001**
ARA/DHA	4.05 ± 1.18^a^	2.96 ± 0.82^b^	2.70 ± 0.95^b^	**<0.001**

1 *Data are means ± SD for parametrically distributed data*.

2 *P-values for overall differences between COGNIS-groups in the ANOVA test (normally distributed variables). Values not sharing the same suffix (a,b,c) were significantly different in the Bonferroni post-hoc test. P < 0.05 are highlighted in bold. SF, Standard infant formula; EF, Experimental infant formula; BF, Breastfed infants; FAs, Fatty acids; ARA, Arachidonic acid; EPA, Eicosapentaenoic acid; AdA, Adrenic acid; DHA, Docosahexaenoic acid; ARA/DHA, Arachidonic acid/Docosahexaenoic acid ratio*.

### Differences in Dietary Intake Between the Three Study Groups

Afterwards, dietary intake was analyzed during the follow-up period (up to 6 years old). Regarding the age of complementary feeding introduction, it was significantly earlier in infant formula groups (SF: 17.06 ± 2.50 weeks; EF: 17.37 ± 2.64 weeks) than in the BF group (22.75 ± 4.91 weeks) (*p* < 0.001). As shown in Supplementary Table 1, significant differences in dietary intake between the three groups were mainly found during the first 18 months of life. In fact, formula-fed infants at 6 months of life showed higher proteins, carbohydrates (CHO), and n-3-PUFA intake in terms of g/day, and calcium, iron, and zinc in terms of mg/day compared to BF infants (all *p* < 0.001). However, daily intake of total lipids (*p* < 0.001) and various LC-PUFAs, including α-linolenic acid (LNA), EPA, and docosapentaenoic acid (DPA) (*p* < 0.001, *p* = 0.024, and *p* < 0.001, respectively), were higher in the BF group compared to SF and EF infants. It is also important to note that ARA and DHA daily intake were different between the three groups, being higher in those infants who were breastfed and lower in those who received SF (both *p* < 0.001).

At 12 months of life, BF infants presented higher total lipids and LNA intake than SF infants (*p* = 0.001 and *p* = 0.022, respectively). Furthermore, higher linoleic acid (LA) and *n*−6-PUFA intake were found in infants fed with EF compared to BF infants (*p* = 0.024 and *p* = 0.004, respectively). Interestingly, EF and BF infants presented similar ARA and DHA daily intake, but had higher intake than SF infants (both *p* < 0.001). Conversely, formula-fed infants showed lower daily intakes of EPA and DPA but higher n-3 PUFAs intakes compared to those who were breastfed (all *p* < 0.001). Regarding minerals, EF infants presented higher calcium daily intake than BF ones (*p* = 0.016), and both formula-fed groups showed higher intake of iron and zinc compared to BF group (all *p* < 0.001; Supplementary Table 1).

Our analysis also showed that participants significantly differed in daily intake of essential macronutrients at 18 months of life. In this regard, daily energy intake was higher in EF-fed infants compared with BF-fed infants (*p* = 0.047). In addition, their protein intake was higher compared to both SF and BF groups (*p* = 0.001). Regarding total lipid intake, the analysis revealed that SF infants showed lower intake than EF and BF infants (*p* = 0.046). However, carbohydrate (CHO) intake was significantly higher in both formula groups in comparison with the BF group (*p* = 0.002). Significant differences were also found for the daily intake of specific PUFAs. Overall, *n*−3-PUFA intake was higher in infants fed with EF compared with BF infants (*p* = 0.021), without differences in n-6 PUFAs between study groups. Particularly, both ARA and DHA intake remained higher in EF and BF infants compared to SF infants (both *p* < 0.001). Daily EPA intake was lower in formula-fed infants in contrast to the BF group (*p* < 0.001), and DPA intake was lower in the SF group with respect to the BF group (*p* = 0.007). Furthermore, higher daily intake of iron and zinc were found in EF infants in comparison with BF infants (*p* = 0.003 and *p* = 0.001, respectively) (Supplementary Table 1).

It is important to note that at 2.5 and 6 years old, no differences were found regarding dietary intake between the three groups. Nevertheless, at 4 years old, we only found differences in total energy (kcal/day) and n-3-PUFA intake being higher in the SF children than in the BF ones (*p* = 0.043 and *p* = 0.022, respectively) (Supplementary Table 1).

### Relationships Between Fatty Acid Status During the First 18 Months of Life and Brain Structure at 6 Years Old

Using stepwise linear regression analysis, we next evaluated potential long-term influence of FA concentrations during the first 18 months of life on later brain structure (brain volume and cortical thickness) at 6 years (Table 6). In this regard, we found that both DHA levels and ARA/DHA index during early life were strongly correlated to later brain volume, mainly in the right parietal and right precuneus regions. In fact, our analysis showed a positive association between the right parietal volume at 6 years old and DHA concentrations presented in infants at 6, 12, and 18 months of age, while ARA/DHA index at these ages were negatively related to right parietal volume later in life (all *p* < 0.001). A positive association was also found between later right parietal volume and ARA levels at 6 months (*p* = 0.021).

**Table 6:** Potential long-term effects of fatty acids status during the first 18 months of life on children's brain structure at 6 years old^a^.

	**Fatty acids**							
**Brain regions**	**6 months**				**12 months**		**18 months**	
**Volume (cm^**3**^)**	**ARA**	**EPA**	**DHA**	**ARA/DHA**	**DHA**	**ARA/DHA**	**DHA**	**ARA/DHA**
Right parietal	β = 0.176 *P* = **0.021**	N/S	β = 0.330 *P*<**0.001**	β = −0.374 *P* < **0.001**	β = 0.341 *P* < **0.001**	β = −0.291 *P* < **0.001**	β = 0.295 *P* < **0.010**	β = −0.313 *P* < **0.001**
Left orbital	N/S	N/S	N/S	N/S	N/S	N/S	N/S	N/S
Right postcentral gyrus	N/S	N/S	β = 0.261 *P* = **0.021**	β = −0.344 *P* = **0.002**	N/S	N/S	N/S	N/S
Right precuneus	β = 0.203 *P* = **0.039**	N/S	β = 0.340 *P* = **0.001**	β = −0.385 *P* < **0.001**	β = 0.350 *P* = **0.001**	β = −0.249 *P* = **0.013**	N/S	β = −0.350 *P* = **0.001**
**Cortical thickness (mm)**								
Left circular insular sulcus	N/S	β = 0.315 *P* = **0.012**	N/S	N/S	N/S	N/S	N/S	N/S
Left occipito–temporal sulcus	N/S	N/S	N/S	N/S	N/S	N/S	N/S	N/S
Right postcentral sulcus	N/S	N/S	N/S	N/S	N/S	N/S	N/S	N/S

a *P-values for linear regression analysis. P-values < 0.05 are highlighted in bold*.

*ARA, Arachidonic acid; EPA, Eicosapentaenoic acid; DHA, Docosahexaenoic acid; ARA/DHA, Arachidonic acid/Docosahexaenoic acid ratio; β, Beta coefficient; N/S, Not significance*.

Similar associations were also found between later right precuneus volume and FA concentrations analyzed in early life. Thus, volume in this brain region exhibited a positive association with ARA levels at 6 months (*p* = 0.039) and DHA levels at 6 and 12 months (both *p* = 0.001), while ARA/DHA index at 6, 12, and 18 months showed negative association with the volume of right precuneus at 6 years old (*p* < 0.001, *p* = 0.013, and *p* = 0.001, respectively). Finally, right postcentral gyrus volume in children aged 6 years was positively related to DHA levels (*p* = 0.021), but negatively associated with ARA/DHA index (*p* = 0.002) at 6 months.

Regarding cortical thickness, our analysis showed a positive association between thickness of the left circular insula and EPA levels in cheek cell glycerophospholipids at 6 months of age (*p* = 0.012).

### Long-Term Influences of Early Nutrient Intake on Children's Brain Structure at 6 Years Old

To further explore the long-term effects of early nutrition on brain structure, we also performed stepwise linear regression analyses that included brain regions which were different between study groups as dependent variable, both nutrients with which daily intake was significantly different, and several confounder variables *(maternal age, familiar socioeconomic status, smoking during pregnancy, age, and sex of the children)* as predictors. The significant associations obtained are shown in Table 7.

**Table 7:** Long-Term influence of early dietary intake on brain structure in children aged 6 years^a^.

	**Brain regions**					
	**Volume (cm** ^**3**^ **)**				**Cortical Thickness (mm)**	
**Macronutrients**	**Right parietal**	**Left orbital**	**Right post-central gyrus**	**Right precuneus**	**Left circular Insular sulcus**	**Right post-central sulcus**
**6 months**						
Lipids (g/day)	N/S	β = −0.237	N/S	N/S	N/S	N/S
		***P*** **=** **0.006**				
ARA (g/day)	β = 0.182	β = −0.222	N/S	β = 0.210	N/S	N/S
	***P*** **=** **0.018**	***P*** **=** **0.015**		***P*** **=** **0.032**		
DPA (g/day)	N/S	β = −0.297	N/S	N/S	N/S	N/S
		***P*** **=** **0.001**				
DHA (g/day)	β = 0.162	β = −0.273	N/S	N/S	N/S	N/S
	***P*** **=** **0.040**	***P*** **=** **0.003**				
Iron (mg/day)	N/S	β = 0.249	N/S	N/S	N/S	N/S
		***P*** **=** **0.007**				
**12 months**						
Lipids (g/day)	N/S	β = −0.294	β = 0.281	N/S	N/S	N/S
		***P*** **=** **0.001**	***P*** **=** **0.009**			
LA (g/day)	N/S	N/S	N/S	N/S	β = 0.242	N/S
					***P*** **=** **0.029**	
LNA (g/day)	N/S	β = −0.322	β = 0.264	N/S	N/S	N/S
		***P*** ** <0.001**	***P*** **=** **0.014**			
ARA (g/day)	N/S	β = −0.264	β = 0.257	N/S	N/S	N/S
		***P*** **=** **0.005**	***P*** **=** **0.019**			
EPA (g/day)	N/S	β = −0.303	N/S	N/S	N/S	N/S
		***P*** **=** **0.002**				
DPA (g/day)	N/S	β = −0.300	N/S	N/S	N/S	N/S
		***P*** **=** **0.001**				
DHA (g/day)	N/S	β = −0.242	β = 0.272	β = 0.237	N/S	N/S
		***P*** **=** **0.012**	***P*** **=** **0.015**	***P*** **=** **0.018**		
*n*−6-PUFAs (g/day)	N/S	N/S	N/S	N/S	β = 0.236	N/S
					***P*** **=** **0.033**	
*n*−3-PUFAs (g/day)	N/S	N/S	β = 0.230	N/S	N/S	N/S
			***P*** **=** **0.033**			
Calcium (mg/day)	N/S	N/S	β = 0.236	N/S	N/S	N/S
			***P*** **=** **0.028**			
**18 months**						
Protein (g/day)	N/S	N/S	β = 0.275	N/S	N/S	β = 0.330
			***P*** **=** **0.013**			***P*** **=** **0.011**
EPA (g/day)	N/S	N/S	N/S	β = 0.218	N/S	N/S
				***P*** **=** **0.027**		
DHA (g/day)	N/S	N/S	N/S	β = 0.208	N/S	N/S
				***P*** **=** **0.036**		
Zinc (mg/day)	N/S	N/S	N/S	N/S	N/S	β = 0.267
						***P*** **=** **0.040**

a *P-values for linear regression analysis. P-values <0.05 are highlighted in bold*.

*CHO, Carbohydrates; LA, Linoleic acid; LNA, α-Linolenic acid; ARA, Arachidonic acid; EPA, Eicosapentaenoic acid; DPA, Docosapentaenoic acid; DHA, Docosahexaenoic acid; PUFAs, Polyunsaturated fatty acids; n−6, Omega-6; n−3, Omega-3; β, Beta coefficient; N/S, Not significance*.

Our results showed that daily intake of certain macro- and micronutrients during early life was positively related to right brain region volume. In this line, we found that right parietal volume showed a positive association with ARA and DHA daily intake at 6 months of life (*p* = 0.018 and *p* = 0.040, respectively), and left orbital volume presented a positive association with iron intake at 6 months of life (*p* = 0.007). Likewise, right postcentral gyrus volume displayed a positive association with lipid intake, LNA, ARA, DHA, n-3-PUFAs, and calcium at 12 months of life (*p* = 0.009, *p* = 0.014, *p* = 0.019, *p* = 0.015, *p* = 0.033, and *p* = 0.028, respectively), and with protein daily intake at 18 months of life (*p* = 0.013). Furthermore, right precuneus volume was positively associated with ARA intake at 6 months of life (*p* = 0.032), DHA intake at both 12 and 18 months of life (*p* = 0.018 and *p* = 0.036, respectively), and EPA intake at 18 months of life (*p* = 0.027).

On the other hand, negative associations were found between the left orbital volume and lipids, ARA, DPA, and DHA daily intake at 6 (*p* = 0.006, *p* = 0.015, *p* = 0.001, and *p* = 0.003, respectively), and 12 months (*p* = 0.001, *p* = 0.005, *p* = 0.001, and *p* = 0.012, respectively). Daily LNA and EPA intake at 12 months of life were also negatively associated with left orbital volume (*p* < 0.001 and *p* = 0.002, respectively).

Regarding cortical thickness, our analysis revealed a positive association between left circular insular sulcus and LA and n-6-PUFA intake at 12 months of life (*p* = 0.029 and *p* = 0.033, respectively). Finally, protein and zinc intake at 18 months were positively related to cortical thickness of the right postcentral sulcus (*p* = 0.011 and *p* = 0.040).

## Discussion

To our knowledge, this is the first study that evaluates long-term impact of an early nutritional intervention based on an infant formula supplemented with several bioactive compounds during the first 18 months of life, on later brain structures, and related neurocognitive function in healthy children aged 6 years. Having in mind that both neurocognitive outcomes and brain structures obtained in all participants are within the normal range, our results seem to show a slightly better neurocognitive performance, particularly in terms of IQ, vocabulary (K-BIT test), and attention (BENCI test) in children fed with the supplemented infant formula compared to those who were breastfed. While long-term effects of early nutrition on neurocognitive development resulted to be lesser than expected, major changes were observed on the brain structure at 6 years old, mainly in children fed with the supplemented infant formula compared to those who received the standard one, and similar to those found in breastfed children. In fact, EF children seem to have greater volumes in the parietal and frontal regions and higher cortical thickness in the insular, parietal, and temporal regions with respect to the SF group (Figure 2). Interestingly, both volumes and cortical thickness in the parietal region were similar among EF and BF groups. Further correlation analyses suggested that EF-related changes in brain structure were positively associated with cognitive performance. In addition to the type of milk feeding received during the first 18 months of life, more detailed nutritional evaluation suggests that proteins, minerals and, fatty acid intake during the first 18 months of life, along with FAs concentrations in cheek cell glycerophospholipids, seem to influence later brain structure. Overall, results obtained here suggest that the intake of an infant formula supplemented with MFGM, LC-PUFAs, synbiotics (nutritional composition closer to breast milk), and key nutrients (FAs, proteins, calcium, iron, and zinc) during the first 18 months of life and the FA status in childhood may influence neurocognitive development and brain structure at least up to 6 years of age.

It is well-established that early nutrition plays a key role on optimal brain structure and function, particularly regarding optimal intake of certain nutrients (proteins, LC-PUFAs, iron, folate, among others) during sensitive periods of brain growth and development (53, 54). Although breastfeeding is the gold standard for infant nutrition during early postnatal life, infant formula intake is currently increasing in low- and high-income countries (55, 56). Consequently, great efforts have been made to enrich or supplement infant formulas with bioactive nutrients found in breast milk to narrow nutritional and functional gaps between both types of infant feeding (13). We have previously reported both short- and long-term positive effects of this infant formula supplemented with bioactive nutrients, including MFGM, LC-PUFAs, and synbiotics, on brain maturation and function assessed as visual function and language development at 18 months of life and 4 years, respectively (27, 57). In addition to these benefits, the current study also suggests that supplemented infant formula seems to have a beneficial impact on child neurocognitive development at 6 years old. In fact, children who received this type of infant formula during their first 18 months of life showed higher scores in IQ, vocabulary, and attention than those who were breastfed, although values obtained were within the normal range in both cases (29, 42). This finding should be doubly discussed. First, better neurocognitive scores obtained in EF children might be partially due to the supplementation of infant formula with bioactive compounds. In this regard, according to the European Food Safety Authority (EFSA) recommendations, our experimental infant formula was supplemented with DHA and ARA (58), which are not only essential for neurogenesis, neuronal migration, and synaptogenesis processes (59), but also have a promising beneficial role on cognitive function and visual acuity (60–63), particularly with higher proportion of DHA and ARA, as well as longer supplementation duration (64). Furthermore, recent studies support the need to supplement infant formulas with DHA together with ARA to achieve optimal plasma concentrations of both fatty acids (14, 65). Moreover, infant formula tested in the COGNIS study was also supplemented with other bioactive nutrients which have separately shown positive impact on neurodevelopment and cognitive function, including MFGM (20–22, 66) and synbiotics (23, 24, 57). However, keeping in mind that each stage of brain development can be affected by different nutrients (67), we cannot determine whether the effects on brain and cognitive function reported here are only related with MFGM, LC-PUFAs, synbiotics or other bioactive compounds supplementation, or, more likely, to their synergistic action.

Secondly, it is important to highlight that the mentioned bioactive compounds are also present in breast milk. Hence, the results obtained here should be considered under controversial relationship between breastfeeding and neurocognitive development at later ages. In fact, although several studies have reported positive effects on neurocognitive and brain development (68–71), others failed to demonstrate long-term effects of breastfeeding on neurocognitive functions (72). This is particularly relevant for child IQ and other cognitive/neurological soft outcomes later in life, on which breastfeeding has little or no positive impact after adjustment for confounding factors including maternal IQ, socioeconomic characteristics, environmental factors (school or parental stimulation), genetic background, and nutritional constituents of breastfeeding (73–77). In the current study, although important potential confounder factors have been considered, we have not been able to control all related factors of social stimulation or nutritional status of the exclusively breastfed infants, including maternal nutritional status, maternal-infant interactions or other physiologic, genetic, or environmental factors that may influence bioavailability, along with status and capacity of LC-PUFAs and other bioactive components that are transferred via breast milk (78–80). Consequently, further studies should be carried out to clarify the role of breastfeeding and the new infant formulas on later cognitive performance, while always keeping in mind that child cognitive development is influenced by a complex mix of genetic and environmental factors, and, probably, by gut microbiota composition and function, as is being recently communicated by different studies (81, 82). However, despite the ideas mentioned above, our findings should not detract from current breastfeeding recommendations (83) because long-term benefits of breastfeeding for both mother and child integral development remain unmatched.

More advanced MRI techniques may offer greater insights about the role of early nutrition on later cognitive performance, identifying both those brain regions involved in neurocognitive development (84) and structural and functional effects of certain nutrients on brain development. In fact, rather than sole nutrient composition, better brain development seems to be associated with whole formula composition, achieving better results in those supplemented or enriched with different bioactive and/or essential nutrients such as LC-PUFA, iron, choline, sphingomyelin, and folic acid (85). In this line, also in accordance with above mentioned neurocognitive effects, our results suggest that early intake of MFGM, LC-PUFAs, and synbiotics through a supplemented infant formula seems to be related to in-depth changes in brain structure development compared to children fed with standard infant formula. Thus, having in mind once more that values obtained are within normal range, EF children showed higher volumes than SF children in different brain regions localized in the parietal lobe which is closely related to attentional and perceptual processes and linguistic functions (86, 87). Additionally, significant differences were also observed in cortical thickness between both formula groups, obtaining higher values in EF children in the inferior circular insula (associated with socio-emotional development) (88, 89), in the occipito-temporal sulcus (related to lexically capacities) (90, 91), and in the postcentral sulcus (related to sensorimotor functional organization) (92). Interestingly, minor differences in both brain volume and cortical thickness were found between EF and BF children, although the latter presented lower volume in the left orbital region which is associated with emotion, attention, inhibition, and memory processes (93–96). Further analysis of our data seems to suggest that aforementioned brain structural differences are related to child neurodevelopment. As a matter of fact, greater brain volumes were correlated to better brain function in terms of executive function and language development. According to Pietschnig et al. larger brain volume has been linked to better cognitive performance across different ages (97). This brain growth has been attributed to axonal density, myelination, and/or increases of fiber diameter (98). In this line, our findings might suggest an accelerated brain development in children fed with the new EF compared to those fed a SF, although further long-term studies are needed to confirm these results.

In addition, to better understand the neurobiological basis of child neurodevelopment, findings obtained so far also seem to support the potential impact of early and later nutrition on neurodevelopment. Overall, after adjusting for confounder variables, our results showed that brain structure later in life might be associated with FA status and protein, mineral, and fatty acid intake during the first 18 months of life. Regarding FAs, among other nutrients, it is well-established that brain myelination process requires both optimal LC-PUFA intake and tissue concentrations (99). In addition, infant brain and subsequent cognitive development is partly affected by the myelination of neural networks (100, 101). In order to determine this interaction, both FA intake analysis and FA profile present in cheek cells were performed. In this line, different studies have observed that dietary fat intake is reflected in the fatty acid composition of the brain and some region-specific differences (102). Furthermore, previous studies have found cognitive differences in children with LC-PUFA supplementation and pointed out a long-term beneficial effect of early life DHA in equilibrium with ARA supplementation, specifically in the attention and inhibition systems and structural, functional, and neurochemical neuroimaging 8 years after supplementation ended (17). Furthermore, during their first 18 months of life, EF infants presented higher DHA, AdA, and ARA concentrations in cheek cell glycerophospholipids and FA intake than SF infants, but no significant differences with respect to the BF group were found. Although it is known that fat quality, rather than its total amount, plays a key role on infants' long-term health outcomes (80), findings obtained in the present study reflect negative associations between the left orbital region volume and FA intake. In fact, BF infants showed higher FA intake and lower volume in this region, along with worse execution in the attention task (more errors of commission in a performance continuous attention task). This association should be interpreted with caution. Since BF is the gold standard, the smaller left orbital region may indicate a different growth pattern of the brain (slower growth) in these children. However, this finding might also suggest potential influence of other factors on brain structure and function, apart from other bioactive components present only in human milk, including physical activity, sleep patterns, or scholar and family routines, that are worthy of further study (103). Unfortunately, we cannot confirm a long-term positive role of BF in the present study. Nevertheless, it seems that BF promotes a better LC-PUFA status and is able to determine a specific pattern of brain development different from that found in formula-fed children, even in those EF with similar LC-PUFA status.

Dietary intake analysis also showed that BF infants presented lower mineral intake (calcium, iron, and zinc) up to 18 months of life compared to formula-fed infants. Likewise, we found positive associations between the abovementioned mineral intake and the left orbital and right postcentral volumes and right postcentral sulcus cortical thickness. Due to their key role on brain development (104), we hypothesized that neurocognitive and brain structure outcomes obtained in BF children aged 6 years might be partly influenced by lower intakes of iron, calcium, and zinc. In fact, iron is essential for neurogenesis processes, neurotransmitter synthesis, brain growth, and dendrite density. Consequently, its deficiency during childhood has been associated with short- and long-term deficiencies in cognitive, motor, socio-emotional, and behavioral development (105, 106). Iron is present in small amounts in human milk (0.03 mg/100 ml) (107) although it has greater bioavailability in relation to infant formulas (108). However, exclusive breastfeeding maintained after 6 months of age is associated with increased risk of iron deficiency. If complementary feeding does not meet this need, iron supplementation is required in some cases (105, 109). Regarding infant formulas, the amount of iron contained in them is sufficient to meet the requirements (58, 110). Additionally, calcium participates in the production of neurons and glial cells (111). Its content in human milk is 32 mg/100 ml, while its content in infant formulas varies between 33.5 and 93.8 mg/100 ml (112), which could explain the lower content in breastfed infants compared to formula-fed ones. Finally, zinc is essential for child growth and development (113). Its supplementation improves motor development and cognitive performance, especially reasoning capacity (114, 115). Its content in human milk is 0.17 mg/100 ml, while its content in infant formulas ranges from 0.34 to 1.01 mg/100 ml (112).

The main strength of this study is its design as a prospective, randomized, double-blind longitudinal study. The COGNIS study is the second study in the world in healthy term infants that includes a long-term follow-up (until 6 years of age) and neuroimaging examination. In the current study, we aimed to demonstrate the long-term effects of early nutrition on brain structure and its consequences on neurodevelopment. To achieve this aim, cognitive function was evaluated using wide range of valid, reliable, and age-appropriate tests focused on diverse child brain domains (43, 44) in contrast with other studies based on evaluation of a single brain domain. Interestingly, our nutritional intervention was performed from 0 to 2 months and prolonged up to 18 months of life, which yields results with added value with respect to other studies with a shorter time of intervention. The experimental infant formula was supplemented with a set of functional nutrients (including MFGM, LC-PUFAs, and synbiotics), thereby providing added value compared to other studies aiming to demonstrate the effect of a single bioactive nutrient. Moreover, it is well-known that neurodevelopment and brain structure in children are influenced by several environmental factors such as nutrition, gender, maternal education, or socioeconomic status, among others (116–119). Thereby, although it is difficult to control all factors involved in child neurodevelopment, several confounding factors previously pointed out (50–52) were taken into account in the statistical analysis performed in the present study in order to obtain consistent results and conclusions.

Nonetheless, the current study has a series of limitations that must be considered. At the beginning of the study, there were no differences between infant formula groups regarding baseline characteristics (27). However, there were differences in parents' age, educational level, and socioeconomic status in the BF group with respect to infant formula groups. The present study has taken those differences into account to carry out the statistical analysis, given that it is well-known that those confounding factors may influence neurodevelopment. On the other side, our study groups are relatively small, not only because of the drop-outs along the long-term follow-up, but also because MRI scanning requires children to remain still. Thus, these results should be interpreted with caution. Moreover, in our study, an individual analysis of each infant's breast milk composition during follow-up has not been performed, and the nutrient intake in the BF infants was estimated based on a complete mature human milk composition reported in the United States Department of Agriculture (USDA) National Nutrient Database for standard reference (107). Furthermore, *p* < 0.05 was used as the statistical threshold, but in the structural analysis, corrections for multiple comparisons were not applied to multiple tests. The current work is an exploratory study, and hence, future studies will be necessary to confirm or refute these results using larger sample size.

In conclusion, our findings suggest that MFGM component-, LC-PUFA-, and synbiotics-supplemented infant formula might be associated to beneficial long-term effects on neurocognitive development and brain structure in terms of brain volumes and cortical thickness in children aged 6 years. These results bring us closer to understand the effects of an adequate nutrition during the first years of life on later brain development and its neuropsychological effects. Analysis of brain structure could provide new knowledge about neural structure underlying neurocognitive function and origins and progression of brain and mental disorders. Therefore, the present study would open future opportunities to develop prevention strategies against brain and mental disorders based on ensuring adequate and individualized nutrition during the first 18 months of life.

## Data Availability Statement

The raw data supporting the conclusions of this article will be made available by the authors, without undue reservation.

## Ethics Statement

The studies involving human participants were reviewed and approved by Research Bioethical Committee from the University of Granada (Spain), as well as the Bioethical Committees for Clinical Research from San Cecilio University Clinical Hospital and University Mother-Infant Hospital of Granada (Spain). Written informed consent to participate in this study was provided by the participants' legal guardian/next of kin.

## Author Contributions

CC is the principal investigator, responsible for the design and coordination of the research, obtained the funding, the guarantor of this work and as such, had full access to all the data in the study and take responsibility for the integrity of the data, accuracy of the data analysis, and the final content of the manuscript. AN-R, ED, and NS-V recruited children and collected clinical and nutritional data. JV-R, AC, MM, and AN-R conducted the statistical analyses and discussed the results. AN-R and JG-S wrote the first draft of the manuscript. All authors edited, reviewed, and approved the manuscript.

## Funding

This project has been funded by Laboratorios Ordesa, S.L. Contract University of Granada General Foundation, No. 3349 and SMARTFOODS (CIEN) Contract University of Granada General Foundation, No. 4003, Spanish Ministry of Economy, Industry and Competitiveness. Furthermore, the project has been partially funded by HORIZON 2020 EU DynaHEALTH Project (GA No. 633595).

## Conflict of Interest

RD-C and JJ are employees of Ordesa Laboratories S.L., company that have funded in part the COGNIS project. The remaining authors declare that the research was conducted in the absence of any commercial or financial relationships that could be construed as a potential conflict of interest.

## Publisher's Note

All claims expressed in this article are solely those of the authors and do not necessarily represent those of their affiliated organizations, or those of the publisher, the editors and the reviewers. Any product that may be evaluated in this article, or claim that may be made by its manufacturer, is not guaranteed or endorsed by the publisher.

## Abbreviations

ARA: Arachidonic acid
AdA: Adrenic acid
BENCI: Computerized battery for neuropsychological evaluation of children
BF: Breastfed infants
BMI: Body Mass Index
DHA: Docosahexaenoic acid
DPA: docosapentaenoic acid
EF: Experimental infant formula
EFSA: European Food Safety Authority
EPA: Eicosapentaenoic acid
FAs: Fatty acids
GWG: Gestational Weight Gain
IQ: Intelligent quotient
K-BIT: Kaufman brief intelligence test
LC-PUFAs: Long-chain polyunsaturated fatty acids
LNA: α-linolenic acid
MFGM: Milk fat globule membrane
MRI: Magnetic resonance imaging
PLON-R: Oral language test of Navarra revised.
