# Supplementary material for: Infant Formula Supplemented With Milk Fat Globule Membrane, Long-Chain Polyunsaturated Fatty Acids, and Synbiotics Is Associated With Neurocognitive Function and Brain Structure of Healthy Children Aged 6 Years: The COGNIS Study
Source: Front Nutr. 2022 Mar 9;9:820224. doi: 10.3389/fnut.2022.820224 (PMC8959863; doi:10.3389/fnut.2022.820224)
Supplement: Supplementary file 1 [file Table_1.DOCX]

Supplementary Material

**Supplementary Table 1.** **Dietary intake up to 6 years of life according to COGNIS study groups^1^.**

|  |  | **6 mo**  **(SF=34, EF=35, BF=31)** | | **12 mo**  **(SF=33, EF=36, BF=32)** | | **18 mo**  **(SF=33, EF=35, BF=31)** | | **2.5 y**  **(SF=22, EF=27, BF=30)** | | **4 y**  **(SF=28, EF=29, BF=31)** | | **6 y**  **(SF=35, EF=35, BF=30)** | |
| --- | --- | --- | --- | --- | --- | --- | --- | --- | --- | --- | --- | --- | --- |
|  |  | **X±SD** | ***p^2^*** | **X±SD** | ***p^2^*** | **X±SD** | ***p^2^*** | **X±SD** | ***p^2^*** | **X±SD** | ***p^2^*** | **X±SD** | ***p^2^*** |
| Energy (kcal/day) | SF | 775.47±152.54 | 0.840 | 920.06±168.38 | 0.140 | 1142.69±252.51^a,b^ | **0.047** | 1373.36±256.61 | 0.597 | 1619.20±229.48^a^ | **0.043** | 1643.63±305.09 | 0.448 |
|  | EF | 802.34±238.56 |  | 1.009.66±220.52 |  | 1229.72±236.39^a^ |  | 1356.76±201.96 |  | 1505.09±226.83^a,b^ |  | 1706.11±257.64 |  |
|  | BF | 777.60±103.23 |  | 946.05±179.49 |  | 1095.41±160.94^b^ |  | 1323.11±126.56 |  | 1469.55±242.15^b^ |  | 1625.75±243.11 |  |
| Protein (g/day) | SF | 18.69±6.01^a^ | **<0.001** | 31.76±8.14 | 0.034 | 41.37±7.48^a^ | **0.001** | 52.88±13.12 | 0.406 | 64.61±12.21 | 0.105 | 66.64±15.57 | 0.226 |
|  | EF | 18.00±5.47^a^ |  | 34.90±7.74 |  | 47.27±10.02^b^ |  | 53.86±12.07 |  | 65.93±17.21 |  | 69.65±12.34 |  |
|  | BF | 13.01±3.70^b^ |  | 29.88±7.94 |  | 39.10±7.15^a^ |  | 49.96±9.00 |  | 58.16±15.09 |  | 63.82±12.10 |  |
| CHO (g/day) | SF | 110.99±25.60^a^ | **<0.001** | 133.02±26.03 | 0.051 | 155.43±41.38^a^ | **0.002** | 154.27±24.56 | 0.069 | 171.54±33.65 | 0.096 | 184.31±32.25 | 0.849 |
|  | EF | 117.37±38.93^a^ |  | 142.80±36.18 |  | 158.99±41.96^a^ |  | 147.34±32.92 |  | 154.14±30.15 |  | 180.00±28.64 |  |
|  | BF | 86.21±20.91^b^ |  | 124.19±29.15 |  | 133.08±23.49^b^ |  | 136.95±21.75 |  | 159.97±27.46 |  | 181.23±36.45 |  |
| Lipids (g/day) | SF | 28.53±6.59^a^ | **<0.001** | 28.99±6.69^a^ | **0.001** | 39.50±9.36^a^ | **0.046** | 60.53±14.70 | 0.551 | 74.96±13.20 | 0.092 | 71.09±18.88 | 0.129 |
|  | EF | 28.98±8.90^a^ |  | 33.21±8.55^a,b^ |  | 44.96±10.15^a^ |  | 61.33±11.65 |  | 69.42±15.20 |  | 78.61±18.75 |  |
|  | BF | 42.36±6.25^b^ |  | 36.64±9.40^b^ |  | 45.19±11.60^a^ |  | 63.94±9.88 |  | 66.34±16.44 |  | 71.73±11.21 |  |
| LA (g/day) | SF | 4.33±1.14 | 0.061 | 3.65±1.02^a,b^ | **0.024** | 4.44±1.55 | 0.342 | 5.49±1.76 | 0.688 | 7.38±2.39 | 0.107 | 6.75±2.26 | 0.327 |
|  | EF | 4.46±1.52 |  | 4.24±1.66^a^ |  | 4.85±2.01 |  | 5.33±1.84 |  | 6.56±1.81 |  | 7.60±2.75 |  |
|  | BF | 3.92±0.62 |  | 3.39±1.05^b^ |  | 4.21±1.77 |  | 5.73±1.64 |  | 6.24±2.06 |  | 7.03±2.13 |  |
| LNA (g/day) | SF | 0.40±0.10^a^ | **<0.001** | 0.37±0.10^a^ | **0.022** | 0.45±0.14 | 0.175 | 0.65±0.32 | 0.364 | 0.75±0.15 | 0.467 | 0.79±0.35 | 0.343 |
|  | EF | 0.40±0.13^a^ |  | 0.40±0.13^a,b^ |  | 0.51±0.16 |  | 0.65±0.29 |  | 0.76±0.30 |  | 0.84±0.27 |  |
|  | BF | 0.52±0.07^b^ |  | 0.45±0.13^b^ |  | 0.46±0.18 |  | 0.76±0.37 |  | 0.69±0.26 |  | 0.76±0.18 |  |
| ARA (g/day) | SF | 0.02±0.03^a^ | **<0.001** | 0.03±0.03^a^ | **<0.001** | 0.05±0.03^a^ | **<0.001** | 0.10±0.06 | 0.860 | 0.14±0.14 | 0.579 | 0.11±0.06 | 0.545 |
|  | EF | 0.12±0.04^b^ |  | 0.09±0.03^b^ |  | 0.11±0.06^b^ |  | 0.10±0.05 |  | 0.12±0.09 |  | 0.12±0.07 |  |
|  | BF | 0.23±0.07^c^ |  | 0.11±0.09^b^ |  | 0.10±0.07^b^ |  | 0.11±0.06 |  | 0.11±0.07 |  | 0.11±0.07 |  |
| EPA (g/day) | SF | 0.01±0.04^a^ | **0.024** | 0.01±0.01^a^ | **<0.001** | 0.01±0.02^a^ | **<0.001** | 0.05±0.09 | 0.646 | 0.06±0.13 | 0.882 | 0.06±0.14 | 0.335 |
|  | EF | 0.00±0.00^a^ |  | 0.01±0.01^a^ |  | 0.02±0.02^a^ |  | 0.04±0.08 |  | 0.08±0.12 |  | 0.12±0.21 |  |
|  | BF | 0.19±0.40^b^ |  | 0.06±0.05^b^ |  | 0.05±0.05^b^ |  | 0.07±0.12 |  | 0.07±0.10 |  | 0.11±0.17 |  |
| DPA (g/day) | SF | 0.02±0.06^a^ | **<0.001** | 0.01±0.01^a^ | **<0.001** | 0.01±0.01^a^ | **0.007** | 0.03±0.03 | 0.333 | 0.02±0.02^a^ | **0.048** | 0.03±0.04 | 0.780 |
|  | EF | 0.00±0.01^a^ |  | 0.01±0.02^a^ |  | 0.02±0.02^a,b^ |  | 0.03±0.03 |  | 0.04±0.05^a^ |  | 0.04±0.04 |  |
|  | BF | 0.08±0.02^b^ |  | 0.04±0.03^b^ |  | 0.02±0.02^b^ |  | 0.04±0.04 |  | 0.03±0.03^a^ |  | 0.03±0.03 |  |
| DHA (g/day) | SF | 0.03±0.11^a^ | **<0.001** | 0.03±0.03^a^ | **<0.001** | 0.05±0.03^a^ | **<0.001** | 0.15±0.15 | 0.657 | 0.18±0.19 | 0.591 | 0.17±0.21 | 0.221 |
|  | EF | 0.08±0.03^b^ |  | 0.09±0.05^b^ |  | 0.11±0.05^b^ |  | 0.15±0.18 |  | 0.23±0.23 |  | 0.28±0.31 |  |
|  | BF | 0.23±0.07^c^ |  | 0.12±0.10^b^ |  | 0.14±0.10^b^ |  | 0.19±0.20 |  | 0.19±0.16 |  | 0.25±0.25 |  |
| n-6-PUFAs (g/day) | SF | 4.08±1.14 | 0.549 | 3.39±0.96^a,b^ | **0.004** | 4.00±1.39 | 0.059 | 4.83±1.76 | 0.664 | 6.72±2.62^a^ | **0.047** | 5.78±2.02 | 0.101 |
|  | EF | 4.17±1.46 |  | 4.02±1.67^a^ |  | 4.38±2.00 |  | 4.71±1.72 |  | 5.56±1.54^a^ |  | 6.89±2.46 |  |
|  | BF | 3.89±0.78 |  | 2.67±1.54^b^ |  | 3.33±1.87 |  | 5.11±1.58 |  | 5.54±1.81^a^ |  | 6.29±1.92 |  |
| n-3-PUFAs (g/day) | SF | 0.38±0.10^a^ | **<0.001** | 0.36±0.10^a^ | **<0.001** | 0.46±0.14^a,b^ | **0.021** | 0.64±0.28 | 0.291 | 0.80±0.21^a^ | **0.022** | 0.77±0.36 | 0.097 |
|  | EF | 0.38±0.12^a^ |  | 0.41±0.14^a^ |  | 0.51±0.16^a^ |  | 0.59±0.21 |  | 0.73±0.24^a,b^ |  | 0.90±0.35 |  |
|  | BF | 0.22±0.06^b^ |  | 0.28±0.13^b^ |  | 0.41±0.14^b^ |  | 0.71±0.34 |  | 0.64±0.19^b^ |  | 0.73±0.25 |  |
| Calcium (mg/day) | SF | 613.12±160.47^a^ |  | 629.73±128.17^a,b^ |  | 732.30±185.80 |  | 907.71±276.30 |  | 913.18 ± 231.74 |  | 916.14 ± 178.05 |  |
|  | EF | 658.83±273.38^a^ | **<0.001** | 665.25±184.94^a^ | **0.016** | 845.26±230.25 | 0.068 | 916.85±204.73 | 0.234 | 916.38 ± 230.02 | 0.965 | 930.20 ± 201.62 | 0.704 |
|  | BF | 385.29±105.24^b^ |  | 542.38±205.12^b^ |  | 748.19±225.61 |  | 816.30±246.99 |  | 931.42 ± 354.15 |  | 872.90 ± 320.85 |  |
| Iron (mg/day) | SF | 8.26±1.96^a^ |  | 10.86±2.34^a^ |  | 11.43±3.42^a^ |  | 8.76±3.39 |  | 8.29±1.54 |  | 9.25±3.06 |  |
|  | EF | 8.68±3.20^a^ | **<0.001** | 11.41±3.22^a^ | **<0.001** | 11.73±3.92^a^ | **0.003** | 7.77±2.75 | 0.537 | 8.60±2.35 | 0.853 | 10.22±3.02 | 0.425 |
|  | BF | 2.18±2.71^b^ |  | 7.02±3.42^b^ |  | 8.95±2.90^b^ |  | 7.90±2.45 |  | 8.61±3.05 |  | 9.69±3.16 |  |
| Zinc (mg/day) | SF | 5.25±1.27^a^ |  | 5.70±1.46^a^ |  | 6.48±1.61^a,b^ |  | 6.90±2.63 |  | 6.61±1.31 |  | 7.47±2.74 |  |
|  | EF | 5.52±1.54^a^ | **<0.001** | 6.23±1.67^a^ | **<0.001** | 7.21±1.89^a^ | **0.001** | 6.38±2.06 | 0.238 | 7.19±2.30 | 0.309 | 7.95±2.28 | 0.612 |
|  | BF | 2.43±1.11^b^ |  | 4.30±1.40^b^ |  | 5.51±1.56^b^ |  | 5.91±1.36 |  | 7.36±3.10 |  | 7.36±2.68 |  |

^1^Nutrients intake are mean ± SD.

^2^*P*-values for differences between COGNIS-groups. ANOVA test for normally distributed variables. Values not sharing the same sufﬁx (a,b,c) were signiﬁcantly different in the Bonferroni *post hoc* test. *P*-values < 0.05 are highlighted in bold.

SF: Standard infant formula; EF: Experimental infant formula; BF: Breastfed infants; CHO: Carbohydrates; LA: Linoleic acid; LNA: α-Linolenic acid; ARA: Arachidonic acid; EPA: Eicosapentaenoic acid; DPA: Docosapentaenoic acid; DHA: Docosahexaenoic acid; PUFAs: Polyunsaturated fatty acids; n-6: Omega-6; n-3: Omega-3.
